# A Rare Behavior of Microprolactinoma with Significant Fluctuation in Size During Pregnancy

**DOI:** 10.1016/j.ajmo.2026.100139

**Published:** 2026-07-25

**Authors:** Sevil Ghafarzadeh-Rad, Soheila Valizadeh, Hamidreza Ashayeri

**Affiliations:** a Endocrine Research Centre, Tabriz University of Medical Sciences, Tabriz, Iran; b Research Center for Evidence-Based Medicine, Iranian EBM Centre: A JBI Centre of Excellence, Faculty of Medicine, Tabriz University of Medical Sciences, Tabriz, Iran

## Abstract

• Pregnancy can cause rapid growth in the size of a pituitary microadenoma.
• The size of a pituitary microadenoma can regress after delivery.
• The prolactinoma responsive to cabergoline may show treatment resistance during pregnancy.
• Tumor apoplexy may happen in the case of rapid tumor growth.

## Clinical Scenario

A 32-year-old with a complaint of galactorrhea and amenorrhea. The patient had no signs or symptoms suggestive of acromegaly or Cushing syndrome. At this time, her prolactin was 227 ng/mL (reference range: 4-26), thyroid-stimulating hormone (TSH) of 2.65 mIU/L (normal range: 0.7-5), free thyroxine (FT_4_) of 1.1 ng/dL (normal range 0.8-1.9), follicular-stimulating hormone (FSH) of 5 mIU/mL (normal range: <25), and insulin-like growth factor-1 (IGF-1) of 198 ng/mL (normal range: 88-246). The magnetic resonance imaging (MRI) of the patient revealed a 7 × 6 × 7 mm intrasellar mass in the pituitary gland, and a diagnosis of microprolactinoma (MiP) was made. She received cabergoline, resolving symptoms and infertility and regulating her menstrual cycle. In her 1st pregnancy, cabergoline was stopped and then continued after the 1st trimester. This pregnancy resulted in a healthy neonate without any obstetric complications or warning signs of tumor growth.

In her 2nd pregnancy, the cabergoline was stopped in the 1st trimester and then continued at 0.5 mg per week. She reported visual field defects and headache. Patient's MRI revealed an increased MiP size reaching macroprolactinoma (25 × 19 × 16 mm) despite receiving cabergoline. In the 28th week, the patient reported nausea, complete loss of sight, and a sudden worsening of headache. She had a Glasgow Coma Score (GCS) of 15, a blood pressure of 110/75 mm Hg, and a sodium level of 137 mEq/L (normal range: 135-145 mEq/L). MRI revealed an oval mass with heterogeneous T1 & T2 signal intensity suggestive of tumor apoplexy. The patient didn't consent to neurosurgical intervention. After delivery, her eyesight improved, and the tumor size regressed to MiP. Cabergoline was continued after delivery. She reports a normal menstrual cycle before the 3rd pregnancy.

**Figure 1:**
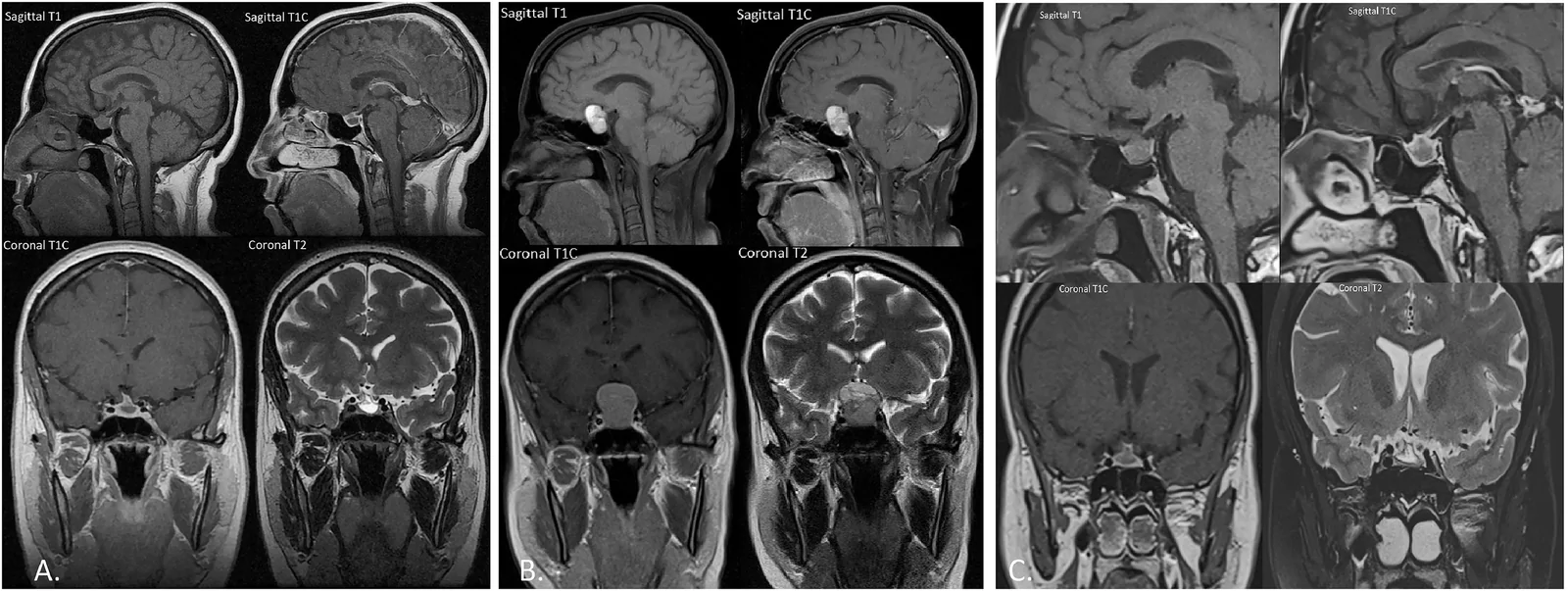
(A) The patient's pituitary MRI before her third pregnancy. (B) Massive enlargement of the tumor compared to section A is seen. (C) Pituitary MRI of the patient after the delivery, compared to section B, shows a regression of tumor size into a microadenoma. T1C; T1-weighted MRI with contrast.

**Figure 2:**
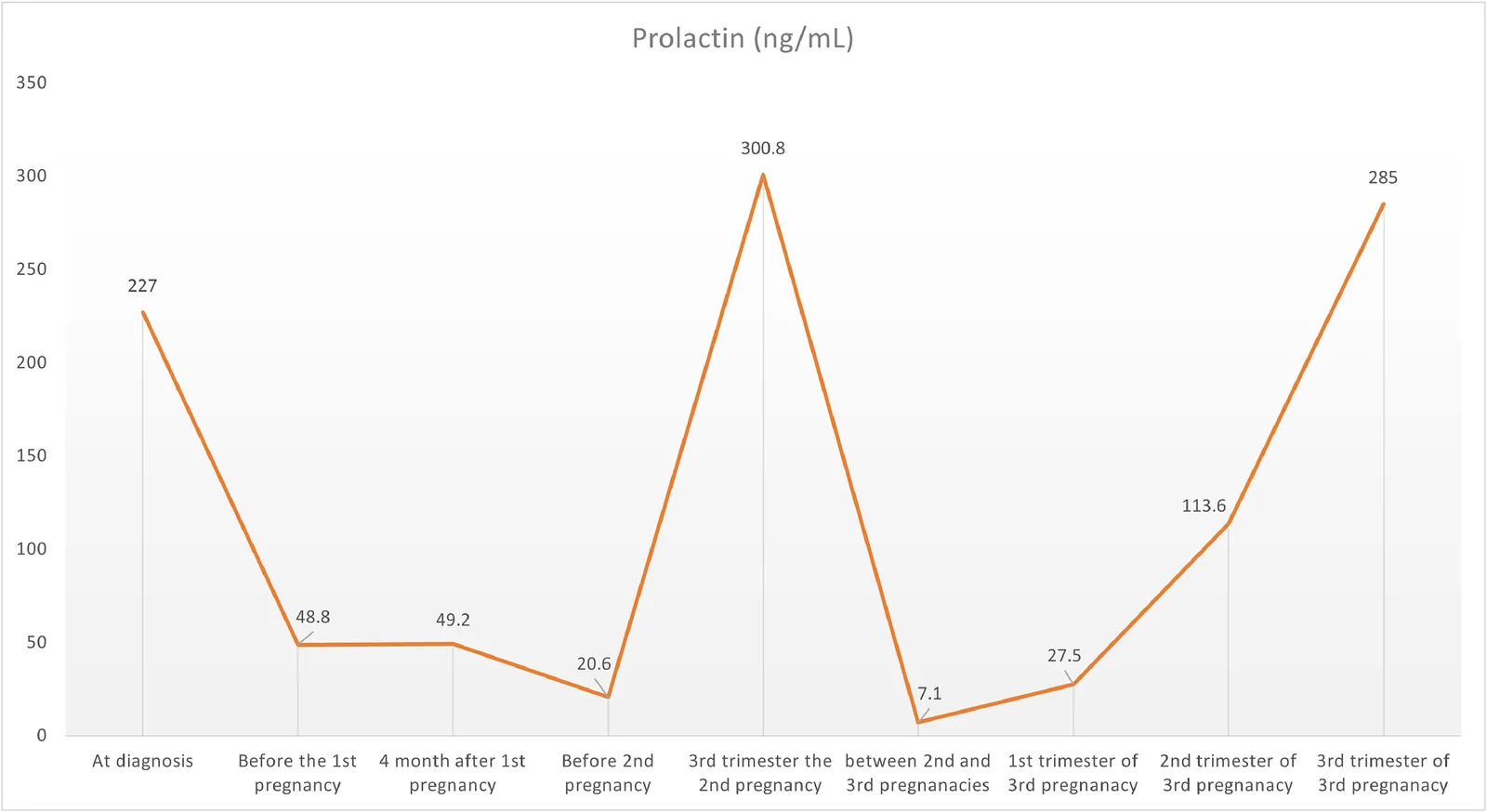
Patient's prolactin levels throughout the pregnancies.

Due to the patient's prior history, cabergoline was continued throughout the 3rd pregnancy. At this time, the patient was 41 years old. She received 1 mg of cabergoline every other day during the 1st trimester and 1 mg every day afterward. No vision problems were reported until the 7th month of the pregnancy. The patient reported blurred vision during the 8th month of pregnancy and was a candidate for surgery, but again didn't consent. Her perimetry study revealed a bitemporal upper quadrantanopia. The blurred vision progressed during the 9th month. Her vision improved after delivery with a gestational age of 37 weeks and 1 day. Figure 1 shows prolactinoma size before the 3rd pregnancy (A), during the 36th week of pregnancy (B), and after delivery (C). Figure 2 shows the prolactin levels throughout the patient's 3 pregnancies.

## Discussion

The chances of symptomatic tumor growth for MiP were reported to be less common (<2%).^1^ The treatment options include reintroduction of a dopamine agonist, delivery of the baby and placenta, and transsphenoidal bulk reduction.^2^ Significant MiP size change during pregnancy is rare but an essential factor to consider in these patients. Patients with pituitary adenomas are also at risk of pituitary apoplexy (PA). PA is a life-threatening condition in which hemorrhage occurs in the pituitary gland, altering the mental status and causing nausea, vomiting, severe headaches, visual field defects, ophthalmoplegia, and signs of panhypopituitarism.^3^ The tumor growth can cause neurological sequelae or pituitary apoplexy, increasing the mortality and morbidity of patients.

## Ethics Approval

This manuscript was approved by the Tabriz University of Medical Sciences ethical committee (IR.TBZMED.REC.1405.065).

## Consent for Participation and Publication

Written informed consent for the publication of any accompanying images was obtained from the patient.

## Availability of Data and Materials

The datasets supporting the conclusions of this article are included within the article and its additional files.

## CRediT authorship contribution statement

**Sevil Ghafarzadeh-Rad:** Writing – original draft, Visualization, Validation, Software, Investigation, Data curation. **Soheila Valizadeh:** Writing – review & editing, Software, Methodology, Investigation. **Hamidreza Ashayeri:** Writing – review & editing, Supervision, Project administration, Methodology, Investigation, Conceptualization.

## Declaration of competing interest

The authors declare that they have no known competing financial interests or personal relationships that could have appeared to influence the work reported in this paper.
